# Coexpression network analysis of gastric adenocarcinoma identifies hub genes as biomarker candidates and their tumor microenvironment associations

**DOI:** 10.1590/1678-4685-GMB-2025-0119

**Published:** 2026-08-10

**Authors:** Ronald Matheus da Silva Mourão, Fabiano Cordeiro Moreira, Jéssica Manoelli Costa da Silva, Daniel de Souza Avelar da Costa, Valéria Cristiane Santos da Silva, Amanda Ferreira Vidal, Leandro Lopes de Magalhães, Ana Karyssa Mendes Anaissi, Samia Demachki, Williams Fernandes Barra, Paulo Pimentel de Assumpção

**Affiliations:** 1 Universidade Federal do Pará, Núcleo de Pesquisas em Oncologia, Belém, PA, Brazil.; 2 Universidade Federal do Pará, Programa de Pós-Graduação em Genética e Biologia Molecular, Belém, PA, Brazil.; 3 Universidade Federal do Pará, Laboratório de Genética Médica e Humana, Belém, PA, Brazil.

**Keywords:** Gastric adenocarcinoma, gene coexpression network, tumor microenvironment, diagnostic biomarkers

## Abstract

Gastric adenocarcinoma (GAC) is characterized by molecular heterogeneity that limits early detection and targeted treatment. We applied weighted gene coexpression network analysis (WGCNA) to paired RNA-seq data from 119 GAC and peritumoral tissue (PTT) samples and identified six coexpression modules with distinct biological identities. Four modules were positively correlated with GAC and two were negatively correlated. Among the 30 hub genes evaluated, several outperformed established clinical biomarkers in accuracy. Specifically, *SPARC* (AUC = 0.89), *COL3A1* (0.87), and *COL1A2* (0.85) exceeded *MUC5AC* (0.76), *VEGFA* (0.68), and *ERBB2* (0.64). Validation in the TCGA-STAD cohort confirmed concordant expression trends for MEblack (5/5 genes) and MEmagenta (4/5), with an overall fold-change correlation of ρ = 0.58 (p = 6.96 × 10⁻⁴). Immune deconvolution delineated two opposing microenvironmental axes, with an adaptive-immune-epithelial program (MEblack) associated with B-cell abundance, and a fibroblast-collagen program (MEmagenta) associated with cancer-associated fibroblast enrichment. DepMap CRISPR screening identified ribosomal hub genes as cell-intrinsic dependencies in gastric cancer cell lines. Among all hub genes, *SPARC*, *COL3A1*, *COL1A2*, *GKN1*, and *GKN2* emerged as the potential biomarker candidates, with *SPARC* additionally showing a validated unfavorable prognostic association in STAD.

## Introduction

Gastric cancer (GC) remains a major global health burden, ranking as the fifth most prevalent cancer with significant incidence and mortality rates affecting both men and women (Bray et al., 2024). Despite a century of progress resulting in declining rates - largely driven by improved diagnosis and the successful eradication of *Helicobacter pylori* - GC remains a clinical challenge (Lin et al., 2024). Alarmingly, the current overall 5-year survival rate for patients is still below 30% (D’Alpino Peixoto et al., 2020). 

A deeper understanding of the molecular and biological basis of GC is therefore needed to improve therapeutic outcomes. Despite substantial progress in uncovering its underlying causes, the heterogeneous nature of GC and the complexity of its tumor biology continue to hinder the development of effective treatments (Joshi and Badgwell, 2021).

Systems biology approaches, particularly weighted gene coexpression network analysis, have provided new tools for studying GC. By constructing weighted gene coexpression networks, it is possible to identify gene modules associated with disease phenotypes, explore functional relationships among genes, and reveal their collective influence on tumor biology. Analyzing these coexpression patterns allows the identification of key gene networks that correlate with specific tumor characteristics and may represent potential therapeutic targets (Li et al., 2021; Zheng et al., 2021; Ding et al., 2023).

This study focuses on constructing and analyzing weighted gene coexpression networks from gastric adenocarcinoma (GAC) and adjacent peritumoral tissue (PTT) samples. By applying a scale-free topology framework, we identified distinct coexpression patterns and explored their associations with tumor characteristics, including the identification of hub genes with central roles within these networks.

The findings characterize gene modules associated with GC and discuss their potential relevance to targeted therapies, aiming to advance a broader understanding of the tumor biology of GC and informing future biomarker development.

## Material and Methods

### Sample characterization and ethical aspects

A total of 119 samples of tumor and PTT were collected from patients diagnosed with the most common type of GC, GAC. The subjects were recruited from the João de Barros Barreto University Hospital in Belém, Brazil. The recruitment of participants and the collection of samples for this study occurred from July 2, 2022, to July 6, 2023. Prior to enrollment, participants were clearly informed of the study objectives and procedures. All participants provided written informed consent. The study was conducted following the Declaration of Helsinki and received approval from the Ethics Committee of the João de Barros Barreto University Hospital (Approval number: 47580121.9.0000.5634).

### Clinical characterization of patients

Of the 119 patients, 81 had complete clinical records available and were subject to comprehensive medical records review and analysis of ancillary clinical data. The review included the following variables: the presence of GAC, pTNM, Laurèn classification, mismatch repair (MMR) status, E-cadherin mutation, and expression of proteins PDL1, PMS2, and MLH1.

### Extraction and quality of the total RNA

Approximately 50-100 mg of tissue from each sample was macerated. Subsequently, 1 ml of TRIZOL^®^ reagent was added to the processed tissue for extraction. Total RNA integrity and concentration were analyzed in the Qubit 4.0 Fluorometer (Thermo Fisher Scientific) and NanoDrop ND- 1000 (Thermo Fisher Scientific). The optimal criteria met for total RNA integrity corresponded to values between 1.8 and 2.2 (A260/A280 ratio), >1.8 (A260/A230 ratio), and RIN (RNA integrity number) ≥ 5. The cutoff accounted for RNA quality variability in clinical samples, balancing cohort representation with data reliability. 

### Construction of cDNA libraries and sequencing

The TruSeq Stranded Total RNA Library Prep Kit with Ribo-Zero Gold (Illumina) was employed to remove cytoplasmic and mitochondrial rRNA. Libraries were then processed using the *NextSeq* ^*®*^ 500 High Output V2 kit - 150 cycles (Illumina) under the conditions specified by the manufacturer. After constructing the libraries, a new integrity assessment was performed on the 2200 TapeStation System (Agilent). The previously created cDNA libraries were loaded into the Illumina NextSeq sequencing system (Illumina) and sequenced using paired-end sequencing.

### Quality, alignment, quantification and transcriptome expression

The initial evaluation of sequencing read quality was conducted using FastQC (v0.11.9). To enhance data integrity, low-quality reads and adapter sequences were eliminated using Trimmomatic, applying a minimum Phred quality score threshold of QV15. This threshold was chosen considering the robustness of Salmon’s k-mer-based pseudoalignment to moderate variations in base quality. Post-filtering, transcript-level quantification was performed with Salmon (v1.5.2), aligning the reads against the human transcriptome reference (hg38). The resulting transcript abundances were imported via Tximport, to construct a DESeq2 object. This object enabled gene expression normalization, accounting for variables such as tissue type (GAC or PTT) and sequencing batch effects. For downstream analyses, variance-stabilized and batch-corrected expression values were utilized.

### Co-expression network construction and hub genes selection

To construct a gene co-expression network, we used the WGCNA package, version 1.73 (Langfelder and Horvath, 2008). Genes with expression levels below one read in more than 50% of the samples were excluded from the analysis to minimize the number of genes with low information content, reduce noise, and avoid artificial correlations. We selected 5,000 genes with the highest variance to prioritize those that are biologically relevant and informative (Table S1). This prioritization ensures that the analysis captures meaningful co-expression patterns associated with key biological processes. The weighted median-based correlation (Biweight midcorrelation) similarity matrix was computed for gene pairs. A soft threshold power value of β = 12 was selected to create a scale-free network. The first principal component of each module’s gene expression was calculated as Module Eigengenes (MEs), and Spearman correlations between MEs and clinical aspects were assessed. Gene significance (GS) for traits and Module Membership (MM) were determined for each gene (Table S2). The grey module comprised genes that did not exhibit correlated expression patterns with any other genes. The filtration of genes within each module was executed based on the following criteria: |GS| > 0.3, |MM| > 0.6, and p<0.05. Network visualization, biological interpretation, and hub identification were performed using Cytoscape v3.7.9. 

Hub genes are defined as those with the highest connectivity degrees, playing central roles in the biological network. Gene’s connectivity can provide significant insights into its functional relevance and impact on disease progression. Hub genes were identified by selecting the top 100 nodes with the highest connectivity within each network; genes with the greatest number of connections among these edges were classified as hubs.

### Gene ontology

To characterize biological pathways and functions associated with each module, genes were subjected to functional enrichment analysis. For this purpose, the Gene Ontology (GO) tool (geneontology.org/) was used via ClusterProfile v4.3.2 (Yu et al., 2012). The enrichment analysis was performed for genes in the modules with a correlation of the absolute values of GS and MM greater than 0.25 (Table S3). We assume that functional enrichment terms with adjusted *p*-values less than or equal to 0.05 are statistically significant. 

### ROC curve

The Receiver Operating Characteristic Curve (ROC) for the expression of each gene was plotted via package pROC v1.18 (Robin et al., 2011). The ROC curve represents the trade-off between sensitivity and specificity across different thresholds, providing insight into the gene’s ability to distinguish between clinical conditions. The Area Under the Curve (AUC) was then calculated to quantify the gene’s diagnostic accuracy, with values near to 1 indicating better performance (Table S4). An AUC > 0.7 is considered acceptable, reflecting the gene’s potential as a clinical biomarker.

### Immune cell deconvolution analysis

To estimate the relative abundance of various immune cell types within bulk RNA-Seq samples, we applied three computational deconvolution methods. Gene expression levels were quantified as Transcripts Per Million (TPM). Cell fractions were estimated using CIBERSORT with the LM22 immune signature matrix, alongside the quanTIseq and EPIC algorithms. Differences in estimated cell proportions between experimental conditions (GAC vs. PTT) were evaluated using the Wilcoxon rank-sum test, applying Benjamini-Hochberg correction for multiple testing (false discovery rate, FDR ≤ 0.05). Furthermore, Spearman correlation analysis was used to explore associations between immune cell fractions that differed significantly between GAC and PTT and the expression levels of hub genes. Key results were visualized using composition bar plots, box plots, and correlation matrices.

### External validation and functional assessment

To validate the generalizability of hub gene expression patterns, we analyzed transcriptomic data from TCGA-STAD (The Cancer Genome Atlas - Stomach Adenocarcinoma project). Expression profiles from TCGA-STAD tumor and PTT samples were compared with our findings to confirm differential expression patterns. Additionally, to assess the functional relevance of hub genes in gastric cancer biology, we interrogated the DepMap portal (Cancer Dependency Map) using CRISPR-based gene-effect scores (Chronos) (Meyers et al., 2017; Dempster et al., 2021). Genes with effect scores below −0.5 were classified as functionally dependent. Finally, pan-cancer prognostic associations were evaluated using the Human Protein Atlas (HPA), which integrates TCGA survival data across cancer types, to assess the translational and prognostic significance of identified hub genes (Uhlén et al., 2015; Thul and Lindskog, 2018).

## Results

### Coexpression network construction and module identification

**Figure 1 -:**
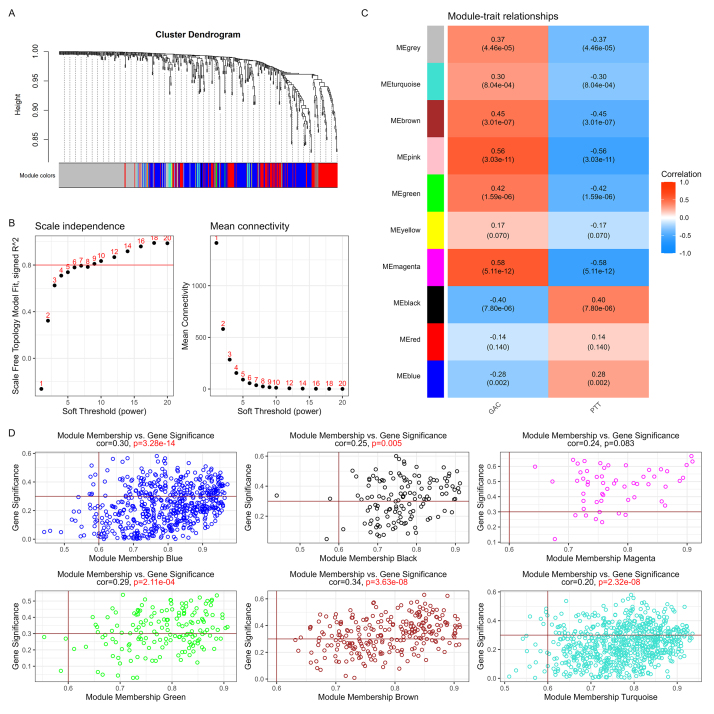
Network construction. (A) Cluster Dendrogram: Hierarchical clustering of genes based on topological overlap to identify distinct coexpression modules. (B) Scale Independence and Mean Connectivity: Plots of the scale-free topology fit index and mean connectivity versus soft-thresholding power, used to select the optimal β = 12. (C) Module-Clinical Relationship: Heatmap showing correlations between module eigengenes and clinical traits (GAC and PTT). Each cell displays the correlation coefficient and corresponding p-value. (D) Gene Significance (GS) vs. Module Membership (MM): Scatter plots depicting the relationship between GS and MM for the six key modules (Blue, Black, Magenta, Green, Brown, and Turquoise).

We compared gene expression between GAC (n = 65) and PTT (n = 54). After variance-stabilizing transformation, the 5,000 most variable protein-coding genes were subjected to WGCNA. Hierarchical clustering at a soft-thresholding power of β = 12 yielded a scale-free topology (Figure 1 A-B ). Correlation of module eigengenes with tissue status identified seven modules with significant and biologically interpretable associations (Figure 1 C ), while the MEgrey module, which contains unassigned genes, was excluded from downstream analyses.

### Coexpression modules positively correlated with GAC

**Figure 2 -:**
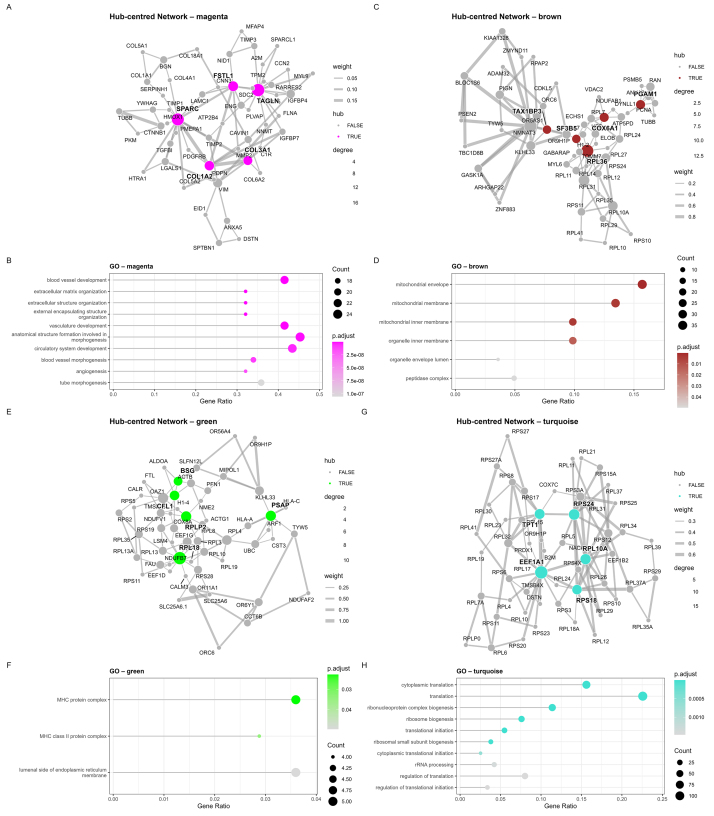
Positively Associated Coexpression Networks and Functional Enrichment Analysis. Hub-centred networks for the Magenta (A), Brown (C), Green (E), and Turquoise (G) modules, where node size reflects connectivity degree and hub genes are highlighted. Accompanying lollipop plots display the top enriched GO terms for module Magenta (B), module Brown (D), module Green (F), and module Turquoise (H), with gene ratio on the x-axis, dot size representing gene count, and color indicating adjusted p-value.

Five modules were positively correlated with GAC (Figure 1 C ), but only four were retained based on significant GS-MM correlation, indicating biologically relevant networks (Figure 1 D ). MEmagenta (55 genes; *r* = 0.58, *p* = 5 × 10⁻¹²) was enriched for extracellular matrix organization, blood vessel development, and angiogenesis (Figure 2 A-B ). Its hub genes, *COL3A1*, *SPARC*, *COL1A2*, *FSTL1*, and *TAGLN*, encode core extracellular matrix (ECM) and vascular remodeling components. MEbrown (253 genes; *r* = 0.45, *p* = 3 × 10⁻⁷) was enriched for mitochondrial envelope and inner-membrane organization (Figure 2 C-D); its hubs *RPL36*, *COX6A1*, *TAX1BP3*, *PGAM1*, and *SF3B5* include components of mitochondrial electron transport. MEgreen (164 genes; *r* = 0.42, *p* = 2 × 10⁻⁶) was enriched for major histocompatibility complex (MHC) class II complex assembly and antigen presentation (Figure 2 E-F ), with hubs *PSAP*, *RPL18*, *BSG*, *CFL1*, and *RPLP2*. MEturquoise (730 genes; *r* = 0.30, *p* = 8 × 10⁻⁴) was enriched for cytoplasmic translation (gene ratio = 74/468) and ribonucleoprotein complex biogenesis (Figure 2 G-H ), with hubs *EEF1A1*, *RPL10A*, *RPS24*, *RPS18*, and *TPT1* forming a highly connected ribosomal network. Although MEpink reached nominal significance (*r* = 0.24, *p* = 0.083), it lacked significant GS-MM correlation (Figure 1 D ) and was excluded from further analysis.

### Coexpression modules negatively correlated with GAC

**Figure 3 -:**
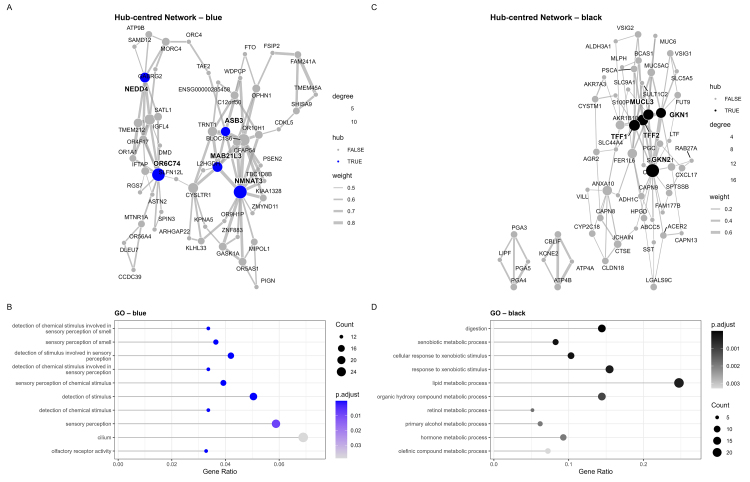
Negatively Associated Coexpression Networks and Functional Enrichment Analysis. Hub-centred networks for the Blue (A) and Black (C) modules, highlighting the most interconnected hub genes. Lollipop plots illustrate the top enriched GO terms for the Blue (B) and Black (D) modules, with gene ratio on the x-axis, dot size representing gene count, and color indicating adjusted p-value.

Two modules were negatively correlated with GAC. MEblue (602 genes; *r* = -0.28, *p* = 0.002) was enriched for olfactory receptor activity and chemical-stimulus detection (Figure 3 A-B ), with hubs *NMNAT3*, *OR6C74*, *MAB21L3*, *ASB3*, and *NEDD4* constituting a network centered on NAD⁺ biosynthesis, ubiquitin-mediated regulation, and stress signaling. MEblack (130 genes; *r* = -0.40, *p* = 8 × 10⁻⁶) was enriched for digestion, xenobiotic-stimulus response, and lipid metabolism (Figure 3 C-D ). Its hub genes, *GKN2*, *TFF2*, *MUCL3*, *TFF1*, and *GKN1*, encode secreted trefoil factors and gastrokines involved in mucus-barrier maintenance, epithelial regeneration, and gastric mucosal homeostasis.

### Hub gene expression and paired tissue analysis

**Figure 4 -:**
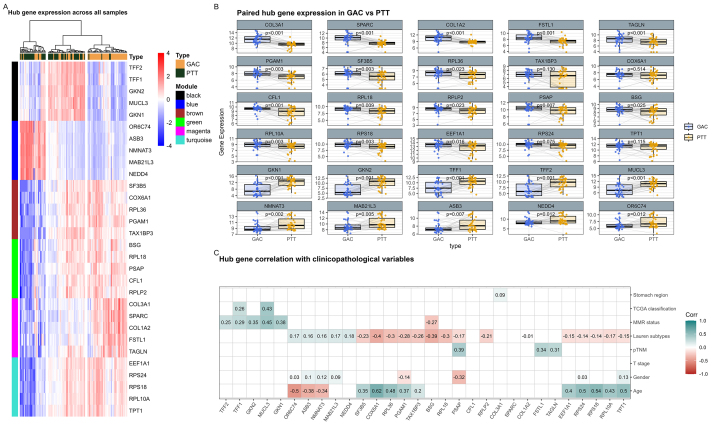
Hub Gene Expression, Paired Comparison, and Clinical Correlations. (A) Heatmap displaying normalized expression of hub genes across all study samples, with rows representing genes (colored by module) and columns representing individual samples (annotated by tissue type). (B) Paired comparison of mean gene expression between GAC and PTT tissues for each hub gene; p-values from Wilcoxon signed-rank tests are shown. (C) Correlation heatmap between hub gene expression and clinicopathological variables, including Lauren classification, pTNM staging, MMR status, T-stage, TCGA classification, gender, and age. Only significant correlations (p < 0.05) are displayed.

Hierarchical clustering of hub gene expression revealed module-specific signatures across the cohort (Figure 4 A ). MEbrown, MEgreen, MEmagenta, and MEturquoise hub genes were predominantly upregulated in GAC, while MEblack and MEblue hubs were downregulated. Principal component analysis showed partial overlap between the GAC and PTT samples (Figure S1), consistent with inter-individual heterogeneity and field cancerization.

Paired analysis controlled for inter-individual variability and sharpened expression differences (Figure 4 B ). All five MEmagenta hub genes were significantly overexpressed in GAC relative to matched PTT (all *p* < 0.001). In MEbrown, *PGAM1*, *SF3B5*, and *RPL36* reached significance (*p* = 0.003, 0.003, and 0.023, respectively), whereas *COX6A1* (*p* = 0.514) and *TAX1BP3* (p = 0.130) did not. All five MEgreen hub genes were significantly overexpressed (*CFL1*, *p* = 0.001; *RPL18*, *p* = 0.009; *RPLP2*, *p* = 0.023; *PSAP*, *p* = 0.007; *BSG*, *p* = 0.025). In MEturquoise, *RPL10A* and *RPS18* were significantly upregulated (both *p* = 0.003), *EEF1A1* showed borderline significance (*p* = 0.016), and *RPS24* (*p* = 0.075) and *TPT1* (*p* = 0.115) did not reach the threshold. All five MEblue hub genes were significantly downregulated in GAC (*NMNAT3*, *p* = 0.002; *MAB21L3*, *p* = 0.005; *ASB3*, *p* = 0.007; *NEDD4*, *p* = 0.012; *OR6C74*, *p* = 0.012). All five MEblack genes showed marked loss of expression (all *p* < 0.001), indicating coordinated suppression of the mucosal defense program in tumor tissue. MEblack and MEmagenta thus showed the most consistent and pronounced expression differences between GAC and PTT, representing the transcriptional alterations in the normal-to-tumor transition.

### Clinicopathological correlations

Spearman correlation analysis between hub gene expression and clinical variables revealed module-specific patterns (Figure 4 C). MEmagenta hub genes *FSTL1* and *TAGLN* correlated positively with pTNM staging (*r* = 0.34, 0.31, respectively), linking their expression to tumor progression and advanced disease burden. MEblack hub genes *TFF1*, *TFF2*, *GKN1*, *GKN2*, and *MUCL3* correlated positively with MMR status (*r* = 0.25-0.45), reinforcing the association between these mucosal defense genes and genomic stability in the normal gastric epithelium. Several MEbrown and MEgreen hub genes (*BSG*, r = −0.39; *COX6A1*, r = −0.40; *RPL18*, r = −0.30; *PGAM1*, r = −0.28) correlated negatively with Laurén classification, indicating differential expression across histological subtypes. MEturquoise hub genes *EEF1A1*, *RPS24*, *RPS18*, *RPL10A*, and *TPT1* were positively correlated with patient age (*r* = 0.40-0.54), suggesting age-dependent upregulation of translational machinery in gastric tissue.

### Immune and stromal cell deconvolution

**Figure 5 -:**
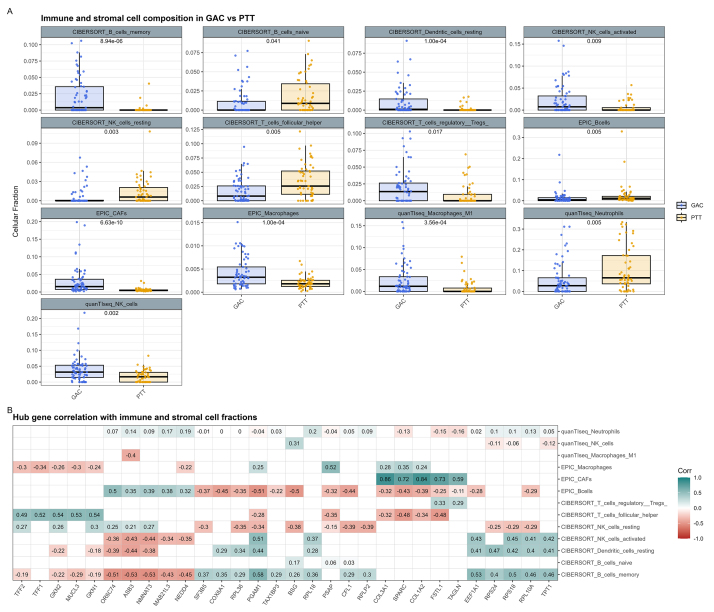
Cellular Deconvolution and Correlation with Hub Genes. (A) Boxplots comparing estimated immune and stromal cell fractions between GAC and PTT using CIBERSORT, EPIC, and quanTIseq algorithms. P-values from Wilcoxon rank-sum tests with Benjamini-Hochberg correction are shown for significantly different cell types. (B) Spearman correlation heatmap between hub gene expression and significantly different immune/stromal cell fractions. Only significant correlations (p < 0.05) are displayed.

Computational deconvolution identified significant shifts in immune and stromal cell composition between GAC and PTT (Figure 5 A). B cells (memory and naive B cells from CIBERSORT, and total B cells from EPIC), resting dendritic cells, activated and resting NK cells, T-follicular helper cells, regulatory T cells, macrophages (from EPIC and M1 from quanTIseq), neutrophils, and cancer-associated fibroblasts differed significantly between tissue types.

Correlation of hub gene expression with deconvolved cell fractions revealed two contrasting microenvironmental programs (Figure 5 B ). MEblack hub genes (*TFF1*, *TFF2*, *GKN1*, *GKN2*, *MUCL3*) correlated positively with T-follicular helper cells (r = 0.49-0.54) and negatively with EPIC macrophages (*r* = −0.24 to −0.34). MEmagenta hub genes (*COL1A2*, *COL3A1*, *SPARC*, *FSTL1*, *TAGLN*) correlated strongly with CAFs (*r* = 0.59-0.86) and negatively with EPIC B cells (*r* = −0.11 to −0.43), with four of five hub genes additionally showing negative correlations with T-follicular helper cells (r = −0.24 to −0.48). MEbrown and MEgreen hub genes, specifically *PGAM1* and *BSG*, correlated negatively with EPIC B cells (*r* = −0.50), while *PGAM1* additionally showed a positive correlation with EPIC macrophages (*r* = 0.25). MEblue hub genes showed significant but opposing associations across B-cell subsets - positively with EPIC B cells (*r* = 0.32-0.51) and negatively with CIBERSORT memory B cells (*r* = −0.43 to −0.53) - alongside negative correlations with activated NK cells (*r* = −0.33 to −0.44), reflecting a heterogeneous immune profile distinct from the adaptive program associated with MEblack.

### Diagnostic performance and benchmarking against established biomarkers

**Figure 6 -:**
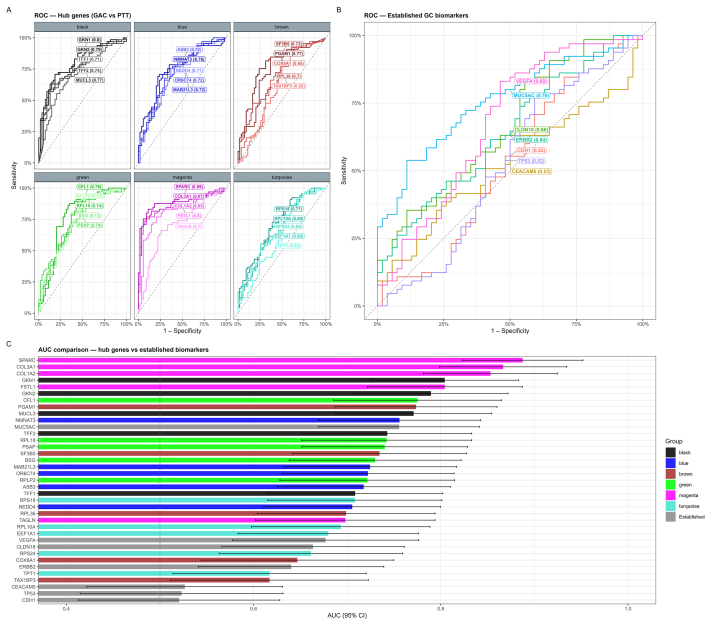
ROC Analysis of Hub Genes and Comparison with Established Gastric Cancer Biomarkers. (A) ROC curves for all 30 hub genes stratified by co-expression module (GAC vs. PTT), with AUC values shown for each gene. (B) ROC curves for a panel of established gastric cancer biomarkers (*MUC5AC*, *VEGFA*, *CLDN18*, *ERBB2*, *CEACAM5*, *TP53*, and *CDH1*) analyzed in the same cohort. (C) Forest plot comparing AUC values with 95% confidence intervals for hub genes versus established biomarkers, ordered by AUC within each group.

ROC analysis evaluated the capacity of each hub gene to discriminate GAC from PTT (Figure 6 A ). MEmagenta hub genes achieved the highest AUC values: *SPARC* (0.89), *COL3A1* (0.87), *COL1A2* (0.85), *FSTL1* (0.80), and *TAGLN* (0.71). MEblack hub genes also performed well: *GKN1* (0.80), *GKN2* (0.79), *MUCL3* (0.77), *TFF2* (0.74), and *TFF1* (0.71). MEgreen hubs ranged from *CFL1* (0.78) to *RPLP2* (0.71). MEbrown showed heterogeneous performance: *PGAM1* (0.77) and *SF3B5* (0.73) performed adequately, while *COX6A1* (0.65) and *TAX1BP3* (0.62) fell below the acceptable threshold. MEblue hubs ranged from *NMNAT3* (0.76) to *NEDD4* (0.71), and MEturquoise from *RPS18* (0.71) to *TPT1* (0.62).

Direct benchmarking against established gastric cancer biomarkers (Figure 6 B-C ) showed that *SPARC*, *COL3A1*, *COL1A2*, *FSTL1*, and *GKN1* each surpassed *MUC5AC* (AUC = 0.76), *VEGFA* (0.68), *CLDN18* (0.66), *ERBB2* (0.64), *CEACAM5* (0.53), *TP53* (0.52), and *CDH1* (0.52). Multiple additional hub genes from MEblack and MEgreen also exceeded *ERBB2*, *CDH1*, and *TP53*, suggesting that module-derived signatures may complement single-marker approaches.

### External validation and functional dependency assessment

**Figure 7 -:**
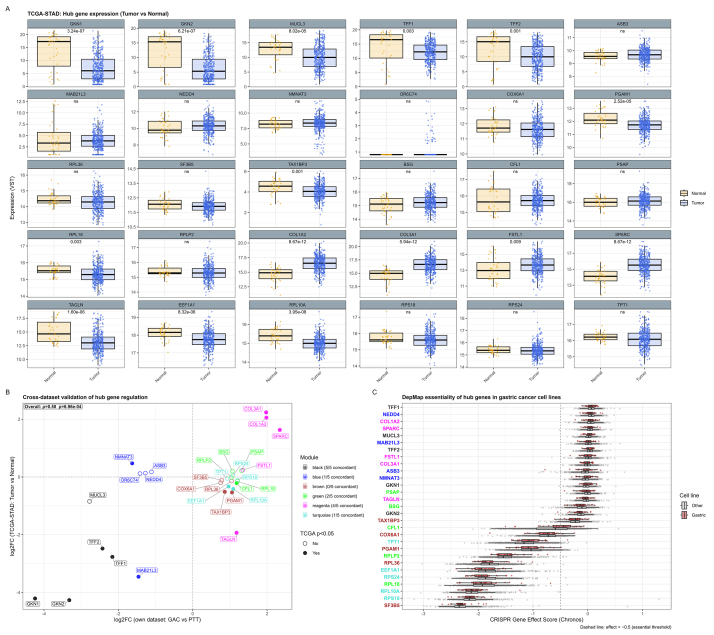
External Validation of Hub Gene Expression and Functional Dependency. (A) Boxplots of hub gene expression in TCGA-STAD tumor and peritumoral normal samples; p-values from differential expression analysis are shown. (B) Cross-dataset comparison of log2 fold-changes between our cohort (x-axis) and TCGA-STAD (y-axis). Each point represents a hub gene, colored by module; filled points indicate significant differential expression in TCGA-STAD (p < 0.05). Overall Spearman correlation: ρ = 0.58, p = 6.96e-04. Concordance rates per module are indicated in the legend. (C) CRISPR gene-effect scores (Chronos) from DepMap for hub genes in gastric cancer versus non-gastric cell lines; the dashed line marks the essentiality threshold (effect < −0.5).

To assess generalizability, we compared hub gene expression in TCGA-STAD tumor versus PTT (Figure 7 A ). MEmagenta genes (*COL3A1*, *p* = 5.94 × 10⁻¹²; *SPARC*, *p* = 8.67 × 10⁻¹²; *COL1A2*, *p* = 8.67 × 10⁻¹²; *TAGLN*, *p* = 1.60 × 10⁻⁶; *FSTL1*, *p* = 0.009) and MEblack genes (*GKN1*, *p* = 3.24 × 10⁻⁷; *GKN2*, *p* = 6.21 × 10⁻⁷; *MUCL3*, *p* = 8.02 × 10⁻⁵; *TFF1*, *p* = 0.003; *TFF2*, *p* = 0.001) were consistently differentially expressed. Additional genes reaching significance included *EEF1A1* (*p* = 8.32 × 10⁻⁶), *RPL10A* (*p* = 3.95 × 10⁻⁸), *RPL18* (*p* = 0.003), *PGAM1* (*p* = 2.52 × 10⁻⁵), and *TAX1BP3* (*p* = 0.001). Fourteen hub genes did not reach significance in TCGA-STAD. Cross-dataset log2 fold-change comparison yielded an overall correlation of *ρ* = 0.58 (*p* = 6.96 × 10⁻⁴), with MEblack showing perfect concordance (5/5 genes) and MEmagenta near-perfect concordance (4/5, with *TAGLN* showing discordant directionality) (Figure 7 B ), confirming that the mucosal defense and ECM-remodeling signatures are the most generalizable across datasets.

CRISPR-based gene-effect scores (Chronos) from DepMap (Figure 7 C ) were available for 29 of the 30 hub genes; *OR6C74* was absent from the database. Of the evaluated genes, eleven from MEturquoise, MEbrown, and MEgreen met the essentiality threshold (effect < −0.5): *SF3B5*, *RPS18*, *RPL10A*, *RPS24*, *RPL18*, *EEF1A1*, *RPL36*, *RPLP2*, *TPT1*, *PGAM1*, and *COX6A1*. Most MEblack and MEmagenta hub genes showed scores near zero. *TFF1*, *TFF2*, and *MUCL3* (MEblack) and *SPARC* and *COL1A2* (MEmagenta) showed positive scores in gastric cancer cell lines.

**Figure 8 -:**
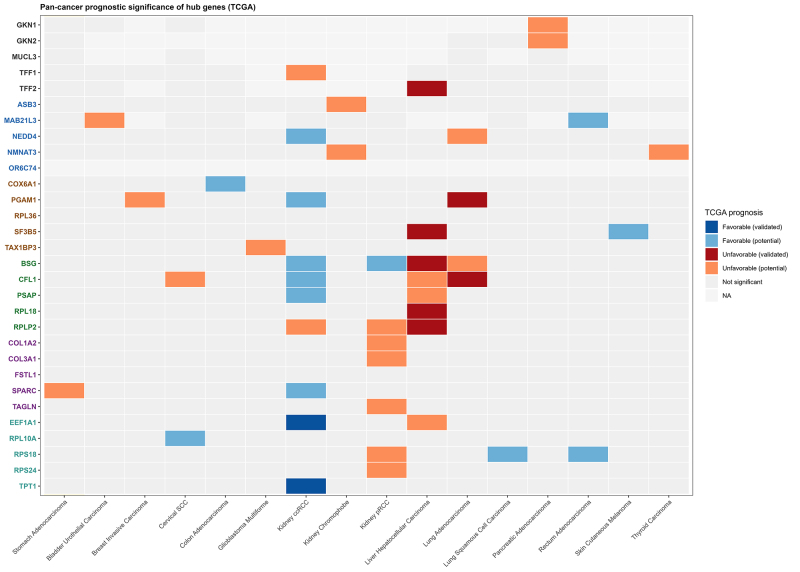
Pan-Cancer Prognostic Significance of Hub Genes (Human Protein Atlas). Heatmap of prognostic associations across 17 TCGA cancer types. Tiles indicate favorable (blue), unfavorable (red), or non-significant (gray) prognosis based on high gene expression. Dark shading indicates validated associations; light shading indicates potential associations. Gene labels on the y-axis are colored by co-expression module.

Pan-cancer prognostic analysis using the Human Protein Atlas (Figure 8) revealed validated prognostic associations for multiple hub genes across cancer types. In Clear Cell Renal Cell Carcinoma (ccRCC), *EEF1A1* and *TPT1* were associated with favorable prognosis. In liver hepatocellular carcinoma, *BSG*, *RPL18*, *RPLP2*, *SF3B5*, *CFL1*, and *PGAM1* all showed validated unfavorable associations, consistent with the essentiality of these ribosomal and metabolic genes identified in DepMap. *TFF2* showed a validated unfavorable association in papillary Renal Cell Carcinoma (RCC), and *MAB21L3* in bladder urothelial carcinoma. *SPARC* was the only hub gene with a validated prognostic signal in stomach adenocarcinoma, associating unfavorable prognosis directly with the MEmagenta ECM-remodeling program; the remaining hub genes lacked STAD-specific validated associations.

## Discussion

The molecular heterogeneity of gastric adenocarcinoma has long complicated efforts to identify reliable diagnostic biomarkers and actionable therapeutic targets (Joshi and Badgwell, 2021). A central difficulty is that tumor and peritumoral tissue often share transcriptomic features, blurring the boundary between normal and neoplastic states. In this study, WGCNA applied to 119 paired GAC and PTT transcriptomes identified six biologically coexpression modules. Across paired expression analysis, clinical correlation, cellular deconvolution, ROC benchmarking, and external validation, two of them, MEblack and MEmagenta, showed the most consistent and informative associations.

In unsupervised clustering (Figure 1 A ), GAC and PTT samples did not segregate into discrete groups but instead intermixed, indicating that global transcriptomic differences between tumor and peritumoral tissues are comparatively narrow. This overlap is consistent with field cancerization, in which histologically normal peritumoral tissue already harbors early molecular alterations (Businello et al., 2021). When paired analysis was applied, comparing GAC and PTT within each patient to control for this variability (Figure 4 B ), expression differences sharpened across all modules, but not uniformly. In MEbrown, *COX6A1* and *TAX1BP3* did not reach significance; in MEturquoise, *RPS24* and *TPT1* did not. Only MEblack and MEmagenta achieved significance across all five hub genes (all p < 0.001), establishing them as the modules with the most coordinated differential expression between GAC and PTT.

Functional enrichment analysis provided a biological framework for these two modules, showing that their hub genes participate in functionally opposing processes. MEblack hub genes, *TFF1*, *TFF2*, *GKN1*, *GKN2*, and *MUCL3*, are secreted trefoil factors and gastrokines that maintain the mucus barrier, promote epithelial regeneration, and protect against acid- and enzyme-induced injury (Chung Nien Chin et al., 2020). Their uniform downregulation in GAC reflects the loss of barrier function as well as the broader silencing of epithelial homeostasis genes, as the MEblack module was also enriched for digestion and xenobiotic response pathways (Figure 3 D ), processes that have been linked to local inflammation and compromised mucosal integrity (Wei et al., 2024). Their positive correlations with MMR proficiency (Figure 4 C ) are consistent with both mucosal defense genes and mismatch repair proficiency being features of a more intact, less transformed gastric epithelium. *GKN2* and *TFF1* have been reported to exert synergistic antiproliferative and pro-apoptotic effects (Kim et al., 2017), while *MUCL3*, a mucin-like protein, may contribute to the integrity of the epithelial-microbial interface, linking mucosal homeostasis to inflammation and tumor biology (Arai et al., 2024).

MEmagenta hub genes, *COL3A1*, *SPARC*, *COL1A2*, *FSTL1*, and *TAGLN*, occupy the opposite pole, as fibrillar collagens, matricellular proteins, and cytoskeletal regulators whose functions converge on ECM deposition, with associated vascular remodeling (Figure 2 A-B ). The ECM not only provides structural support but modulates proliferation, migration, and invasion (Pickup et al., 2014). *COL3A1* and *COL1A2* overexpression has been associated with tumor size and depth of invasion in gastric cancer (Li et al., 2016; Zhou et al., 2022), *FSTL1* with angiogenesis and AKT-pathway activation (Wu et al., 2021), and *TAGLN* with cytoskeletal reorganization and cell motility (Zhou *et al.,* 2016). Their positive correlations with pTNM staging (Figure 4 C) associate them with tumor burden and clinical progression. MEblack and MEmagenta thus define two transcriptional poles, characterized by a preserved epithelial-mucosal state and a remodeled, collagen-dense tumor stroma, respectively.

The remaining modules positively correlated with GAC reflect broader metabolic and immunological reprogramming within the tumor. MEbrown, enriched for mitochondrial membrane organization, includes hub genes involved in electron transport and ribosomal assembly, consistent with the metabolic reprogramming that supports altered bioenergetics in tumor cells (Faubert et al., 2020). MEgreen, enriched for MHC class II complex assembly, points to a tumor-associated antigen presentation program. MEturquoise, the largest module, was dominated by cytoplasmic translation and ribonucleoprotein biogenesis. Hub genes from these three modules correlated negatively with Laurén classification (Figure 4 C ), indicating higher expression in the intestinal subtype relative to the diffuse subtype, which may reflect distinct metabolic and translational demands between these histological categories. MEturquoise hub genes also correlated positively with patient age, consistent with reports of age-associated increases in translational demand (Jiao et al., 2023).

MEblue, the second negatively correlated module, presented a distinct biological profile. Its enrichment for olfactory-receptor activity, driven largely by *OR6C74*, an olfactory receptor gene (Chung et al., 2022), places it at the edge of an unexplored area of cancer biology. Other hub genes in this module, *NMNAT3*, involved in NAD⁺ biosynthesis and p53 modulation (Liu et al., 2021), *NEDD4,* an E3 ubiquitin ligase implicated in tumor suppressor degradation (Sun et al., 2014), and *MAB21L3* (Qin et al., 2024), have documented or putative roles in other malignancies, but MEblue showed weak cross-dataset reproducibility (1/5 concordance in TCGA-STAD) and heterogeneous immune-cell correlations, positioning it as a hypothesis-generating module requiring targeted functional validation.

Cellular deconvolution placed MEblack and MEmagenta in contrasting microenvironmental contexts (Figure 5 B ). MEblack hub gene expression was associated with adaptive immune cell enrichment, particularly T-follicular helper cells, and inversely with EPIC macrophages. MEmagenta hub gene expression was associated with a fibroblast-dominated stroma, evidenced by strong correlations with CAFs and negative correlations with both B cells and T-follicular helper cells, suggesting a broadly immunosuppressive stromal context. MEbrown and MEgreen hub genes, specifically *PGAM1* and *BSG*, correlated negatively with EPIC B cells, with *PGAM1* additionally showing a positive association with EPIC macrophages. MEblue hub genes showed inconsistent associations across B-cell subsets, positive with EPIC B cells but negative with CIBERSORT memory B cells, alongside negative correlations with activated NK cells, likely reflecting algorithm-dependent differences rather than a coherent biological signal. These are co-variation patterns, not causal relationships, but their consistency across three independent deconvolution algorithms is notable. The observation that higher CAF-associated gene expression coincides with lower B-cell abundance raises the possibility that dense ECM imposes physical or paracrine barriers to lymphocyte infiltration (Desbois and Wang 2021) - a question requiring experimental testing.

ROC analysis further assessed the diagnostic utility of these hub genes (Figure 6). In our cohort, the network-derived hub genes (Figure 6 A ) outperformed biomarkers currently used in clinical practice (Figure 6 B). *SPARC* achieved the highest discriminatory capacity (AUC = 0.89), surpassing all conventional markers tested, including *MUC5AC*, *VEGFA*, *ERBB2*, *TP53*, and *CDH1* (Figure 6 C ). MEblack hub genes *GKN1* and *GKN2* also exceeded most established markers. This advantage likely reflects a fundamental difference, since conventional biomarkers capture single-gene alterations, whereas hub genes derive from coordinated multi-gene transcriptional programs whose network-level coherence may underlie their superior discriminatory capacity.

External validation in TCGA-STAD confirmed the robustness of these two signatures (Figure 7). MEblack achieved perfect directional concordance (5/5 genes) and MEmagenta near-perfect concordance (4/5) (Figure 7 A-B ). The single discordant gene, *TAGLN*, encodes transgelin, a smooth-muscle actin-binding protein whose expression may be particularly sensitive to variation in stromal composition between cohorts. The overall fold-change correlation between our cohort and TCGA-STAD was *ρ* = 0.58 (*p* = 6.96 × 10⁻⁴) (Figure 7 B ). Weaker reproducibility for MEbrown (0/5), MEblue (1/5), and MEturquoise (1/5) may reflect greater susceptibility to differences in tissue processing or population-specific expression patterns. Our Northern Brazilian cohort, with high *Helicobacter pylori* prevalence and distinctive dietary and genetic backgrounds, may contribute to context-dependent transcriptional programs not fully replicated in TCGA-STAD.

The DepMap CRISPR data clarified the functional identity of each module. Eleven hub genes from MEturquoise, MEbrown, and MEgreen met the essentiality threshold (effect < −0.5), consistent with tumor-cell dependence on translational and mitochondrial machinery. TFF1, TFF2 and *MUCL3* (MEblack) and *SPARC* and *COL1A2* (MEmagenta) showed positive scores, suggesting that their loss confers a proliferative advantage in gastric cancer cell lines, consistent with the antiproliferative role of MEblack genes in normal gastric epithelium. MEblack and MEmagenta genes thus emerge as candidate diagnostic biomarkers rather than therapeutic targets, while ribosomal and metabolic hub genes represent cell-intrinsic dependencies warranting further investigation.

Pan-cancer prognostic analysis via the Human Protein Atlas (Figure 8) extended these findings, revealing validated associations for *EEF1A1* and *TPT1* (favorable, ccRCC), *BSG*, *RPL18*, *RPLP2*, *SF3B5*, *CFL1*, and *PGAM1* (unfavorable, liver hepatocellular carcinoma), *TFF2* (unfavorable, papillary RCC), and *MAB21L3* (unfavorable, bladder urothelial carcinoma). SPARC showed a validated unfavorable prognostic association in stomach adenocarcinoma - the only hub gene with a STAD-specific signal - directly linking the MEmagenta ECM-remodeling program to clinical outcome in gastric cancer. The breadth of prognostic signals across multiple tumor types indicates that the transcriptional programs captured by our modules reflect broadly conserved oncogenic mechanisms, whose significance in gastric cancer should be evaluated in molecularly stratified cohorts.

This study has limitations. It is single-center, WGCNA identifies coexpression rather than causation, and bulk deconvolution provides estimates rather than direct cell-type measurements. Functional validation through CRISPR perturbation in patient-derived organoids, and single-cell resolution of the tumor microenvironment, represent necessary next steps.

Taken together, our findings suggest that gastric adenocarcinoma transcriptomes are organized along a molecular axis defined by two opposing programs: the mucosal defense signature of MEblack characterizes the intact epithelium, while the stromal remodeling signature of MEmagenta marks the transition to a collagen-dense tumor microenvironment. Both programs demonstrate high reproducibility across cohorts, are embedded in contrasting immune-stromal niches, and offer high diagnostic accuracy compared to conventional biomarkers. Among the 30 hub genes examined, *SPARC*, *COL3A1*, *COL1A2*, *GKN1*, and *GKN2* emerge as key biomarker candidates, supported by transcriptomic, microenvironmental, functional, and prognostic evidence.

## Supplementary material

The following online material is available for this article:

Table S1 - Normalized gene expression. Gene expression values for each sample based on transcript quantification.

Table S2 - GS and MM. Correlation metrics between gene expression and clinical traits.

Table S3 - Ontology. Results of Gene Ontology enrichment analysis for selected gene sets

Table S4 - AUC. Performance evaluation of gene-based classifiers or predictive models using the AUC

Figure S1 - Principal Component Analysis (PCA) differentiating GAC and PTT samples

## Data Availability

 The raw data supporting the conclusions of this article can be made available by the corresponding author upon reasonable request.
