# Supplementary material for: Coexpression network analysis of gastric adenocarcinoma identifies hub genes as biomarker candidates and their tumor microenvironment associations
Source: Genet Mol Biol. 2026 Aug 10;49(Suppl 4):e20250119. doi: 10.1590/1678-4685-GMB-2025-0119 (PMC13474574; doi:10.1590/1678-4685-GMB-2025-0119)
Supplement: Supplementary file 1 [file 1415-4757-GMB-49-s4-e20250119-s5.pdf]

Genetics and Molecular Biology
https://doi.org/10.1590/1678-4685-GMB-2025-0119

Supplementary Material to "Coexpression network analysis of gastric adenocarcinoma identifies hub genes as biomarker candidates and their tumor microenvironment associations"

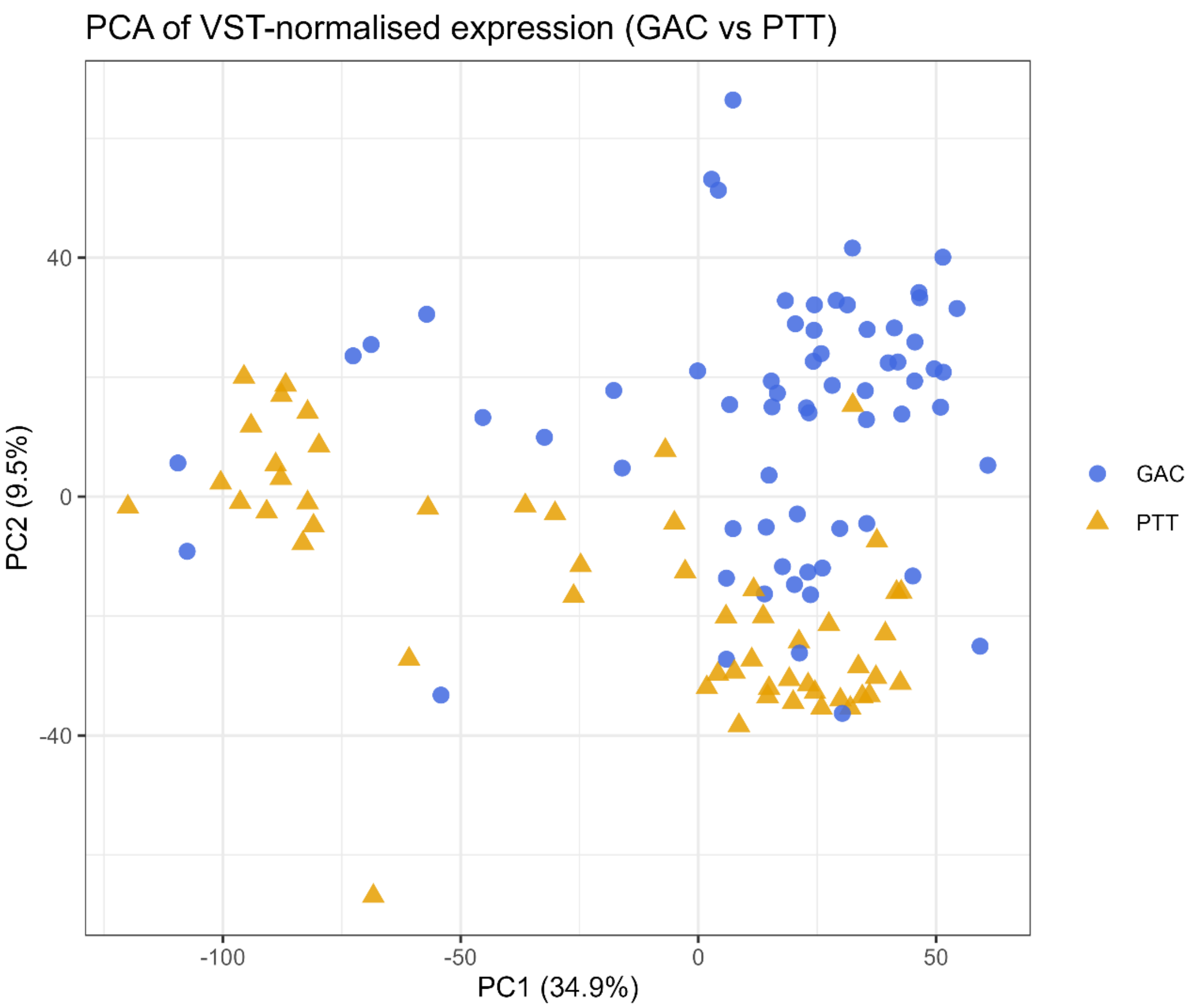

Figure S1 - Principal Component Analysis (PCA) differentiating GAC and PTT samples. Each point represents an individual sample, plotted based on the first two principal components (PC1 and PC2). Samples are color-coded according to their respective tumor groups. The distinct clustering of GAC and PTT samples indicates substantial differences in their molecular characteristics.
