# Supplementary material for: Coexpression network analysis of gastric adenocarcinoma identifies hub genes as biomarker candidates and their tumor microenvironment associations
Source: Genet Mol Biol. 2026 Aug 10;49(Suppl 4):e20250119. doi: 10.1590/1678-4685-GMB-2025-0119 (PMC13474574; doi:10.1590/1678-4685-GMB-2025-0119)
Supplement: Supplementary file 2 [file 1415-4757-GMB-49-s4-e20250119-s1.zip › gmb-2025-0119_suppl1/gmb-2025-0119-legend_table S1.pdf]

Genetics and Molecular Biology
https://doi.org/10.1590/1678-4685-GMB-2025-0119

Supplementary Material to "Coexpression network analysis of gastric adenocarcinoma identifies hub genes as biomarker candidates and their tumor microenvironment associations"

Table S1 - Normalized gene expression. Gene expression values for each sample based on transcript quantification. Rows correspond to individual samples, and columns represent genes. The matrix includes expression levels used in downstream co-expression network analyses.
