# Supplementary material for: Coexpression network analysis of gastric adenocarcinoma identifies hub genes as biomarker candidates and their tumor microenvironment associations
Source: Genet Mol Biol. 2026 Aug 10;49(Suppl 4):e20250119. doi: 10.1590/1678-4685-GMB-2025-0119 (PMC13474574; doi:10.1590/1678-4685-GMB-2025-0119)
Supplement: Supplementary file 3 [file 1415-4757-GMB-49-s4-e20250119-s2.zip › gmb-2025-0119_suppl2/gmb-2025-0119-legend_table S2.pdf]

Genetics and Molecular Biology
https://doi.org/10.1590/1678-4685-GMB-2025-0119

Supplementary Material to "Coexpression network analysis of gastric adenocarcinoma identifies hub genes as biomarker candidates and their tumor microenvironment associations"

Table S2 - GS and MM. Correlation metrics between gene expression and clinical traits. For each gene, Gene Significance (GS) indicates the strength of association with a given trait, while Module Membership (MM) measures the correlation of the gene with its respective co-expression module. These values help identify hub genes with potential biological relevance.
