# Supplementary material for: Coexpression network analysis of gastric adenocarcinoma identifies hub genes as biomarker candidates and their tumor microenvironment associations
Source: Genet Mol Biol. 2026 Aug 10;49(Suppl 4):e20250119. doi: 10.1590/1678-4685-GMB-2025-0119 (PMC13474574; doi:10.1590/1678-4685-GMB-2025-0119)
Supplement: Supplementary file 5 [file 1415-4757-GMB-49-s4-e20250119-s4.zip › gmb-2025-0119_suppl4/gmb-2025-0119-legend_table S4.pdf]

Genetics and Molecular Biology
https://doi.org/10.1590/1678-4685-GMB-2025-0119

Supplementary Material to "Coexpression network analysis of gastric adenocarcinoma identifies hub genes as biomarker candidates and their tumor microenvironment associations"

Table S4 - AUC. Performance evaluation of gene-based classifiers or predictive models using the AUC. Higher AUC values indicate better discriminatory power, useful for identifying genes or signatures with biomarker potential.
